# The genome sequence of the marbled rockcod, *Notothenia rossii *Richardson, 1844

**DOI:** 10.12688/wellcomeopenres.21270.1

**Published:** 2024-04-24

**Authors:** Iliana Bista, Martin Collins, Wellcome Sanger Institute Tree of Life Management, Samples and Laboratory team, Wellcome Sanger Institute Scientific Operations: Sequencing Operations, Wellcome Sanger Institute Tree of Life Core Informatics team, Tree of Life Core Informatics collective

**Affiliations:** 1 LOEWE Centre for Translational Biodiversity Genomics, Frankfurt, Germany; 2 Senckenberg Research Institute, Frankfurt, Germany; 3 Naturalis Biodiversity Center, Leiden, South Holland, The Netherlands; 4 British Antarctic Survey, NERC, Cambridge, England, UK

**Keywords:** Notothenia rossii, marbled rockcod, genome sequence, chromosomal, Perciformes

## Abstract

We present a genome assembly from an individual *Notothenia rossii* (the marbled rockcod; Chordata; Actinopterygii; Perciformes; Nototheniidae). The genome sequence is 1,042.9 megabases in span. Most of the assembly is scaffolded into 12 chromosomal pseudomolecules. The mitochondrial genome has also been assembled and is 21.68 kilobases in length. Gene annotation of this assembly on Ensembl identified 24,432 protein coding genes.

## Species taxonomy

Eukaryota; Opisthokonta; Metazoa; Eumetazoa; Bilateria; Deuterostomia; Chordata; Craniata; Vertebrata; Gnathostomata; Teleostomi; Euteleostomi; Actinopterygii; Actinopteri; Neopterygii; Teleostei; Osteoglossocephalai; Clupeocephala; Euteleosteomorpha; Neoteleostei; Eurypterygia; Ctenosquamata; Acanthomorphata; Euacanthomorphacea; Percomorphaceae; Eupercaria; Perciformes; Notothenioidei; Nototheniidae; *Notothenia*; *Notothenia rossii* Richardson, 1844 (NCBI:txid101497).

## Background

**Figure 1.**
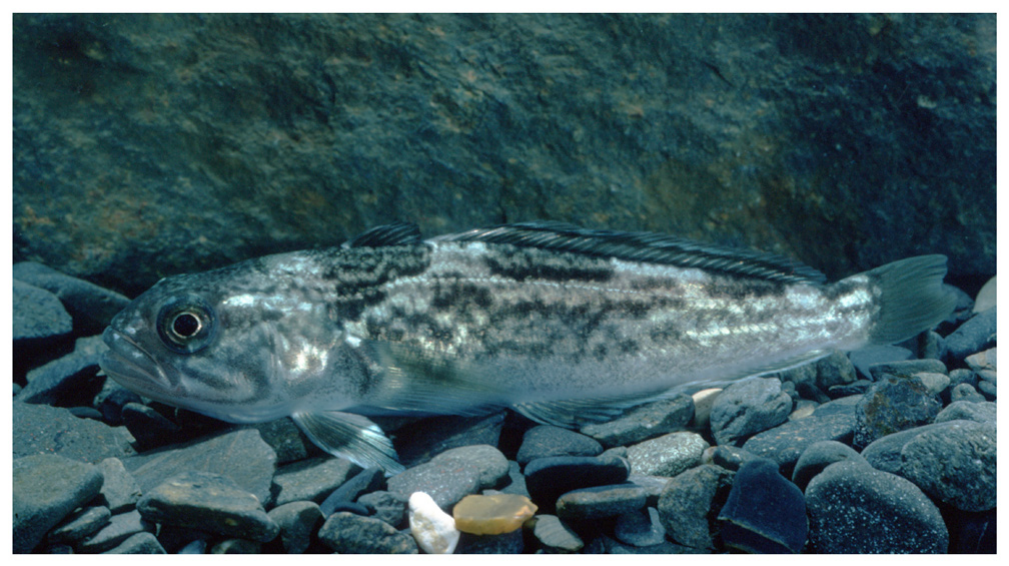
Photograph of *Notothenia rossii* from the British Antarctic Survey (not the specimen used for genome sequencing).

*Notothenia rossii* (marbled rockcod) ( Figure 1) is a member of the notothenioid group (Suborder: Notothenioidei), a well-established fish radiation inhabiting the seas surrounding the Antarctic continent ( Eastman, 2005; Matschiner *et al.*, 2011). The species belongs to the Nototheniidae family, the largest subgroup of the notothenioids comprising 49 species ( Eastman, 2005; Eastman & Eakin, 2021). The notothenioid radiation is characterised by the appearance of antifreeze genes ( *afgp*) ( Chen *et al.*, 1997), which are present in all Antarctic members of the group (cryonotothenioids) including the Nototheniidae, and along with other adaptations enable their survival to extreme cold conditions of the Southern Ocean.

The position of *N. rossii* populations in the water column changes during their life cycle. Adults are bentho-pelagic on the South Georgia shelf and produce large (4–5 mm) planktonic eggs in winter ( Kock *et al.*, 2004). The eggs hatch as planktonic larvae (14–30 mm TL) that develop into a blue pelagic phase (30–70 mm TL) which occupy near-surface waters. The blue phase settle in coastal kelp habitats, where they become demersal and develop a brown colouration ( Burchet, 1983). Juveniles remain in coastal habitats for around five years, until they approach maturity and migrate off shore ( Burchet, 1983; Hollyman *et al.*, 2021), a distribution which also resembles another mostly sympatric species the black rockcod ( *Notothenia coriiceps*) ( Calì *et al.*, 2017). Observed maximum body size for the marbled rockcod is usually close to 80 cm TL ( Hollyman *et al.*, 2021) with maximum reported size reaching 92 cm TL ( DeWitt *et al.*, 1990). Males mature earlier and spawn at smaller body size compared to females ( Hollyman *et al.*, 2021). Feeding behaviour includes a large variety of prey making this species a generalist feeder, with prey including many groups such as various fish, and crustaceans like hyperiids, mysids, amphipods, and euphausiids (krill) ( Hollyman *et al.*, 2021).

Historically *N. rossii* was the first major target of the fishing industry in the Southern Ocean which heavily exploited its populations, eventually leading to a ban on targeted fishing for *N. rossii* in 1985, when the Commission for the Conservation of Antarctic Marine Living Resources (CCAMLR) applied conservation measures for its protection ( Kock *et al.*, 2004). Recovery of fish populations after overfishing events can be influenced by oceanographic, life history traits, and connectivity, with *N. rossii* populations still struggling to recover. Though there is a high degree of population connectivity, the poor retention of egg and larval stages during planktonic phases, and increased mortality due to predator pressure (fur seals) potentially hinders recovery of *N. rossii* populations ( Young *et al.*, 2012).

Here we present a genome assembly generated using a juvenile specimen collected using a Neuston net from the Antarctic Polar Front area, during the JR19001 cruise onboard the James Clark Ross research vessel. This chromosomal assembly of *N. rossii*, provides an improvement on our previous assembly for this species (fNotRos1, PRJEB53175, GCA_943590865.1 ( Bista *et al.*, 2023)).

## Genome sequence report

The genome was sequenced from a *Notothenia rossii* collected from the Southern Ocean (–54.42, –45.88). A total of 24-fold coverage in Pacific Biosciences single-molecule HiFi long reads was generated. Primary assembly contigs were scaffolded with chromosome conformation Hi-C data. Manual assembly curation corrected 58 missing joins or mis-joins and removed 4 haplotypic duplications, reducing the scaffold number by 2.58%, and increasing the scaffold N50 by 99.18%.

**Figure 2.**
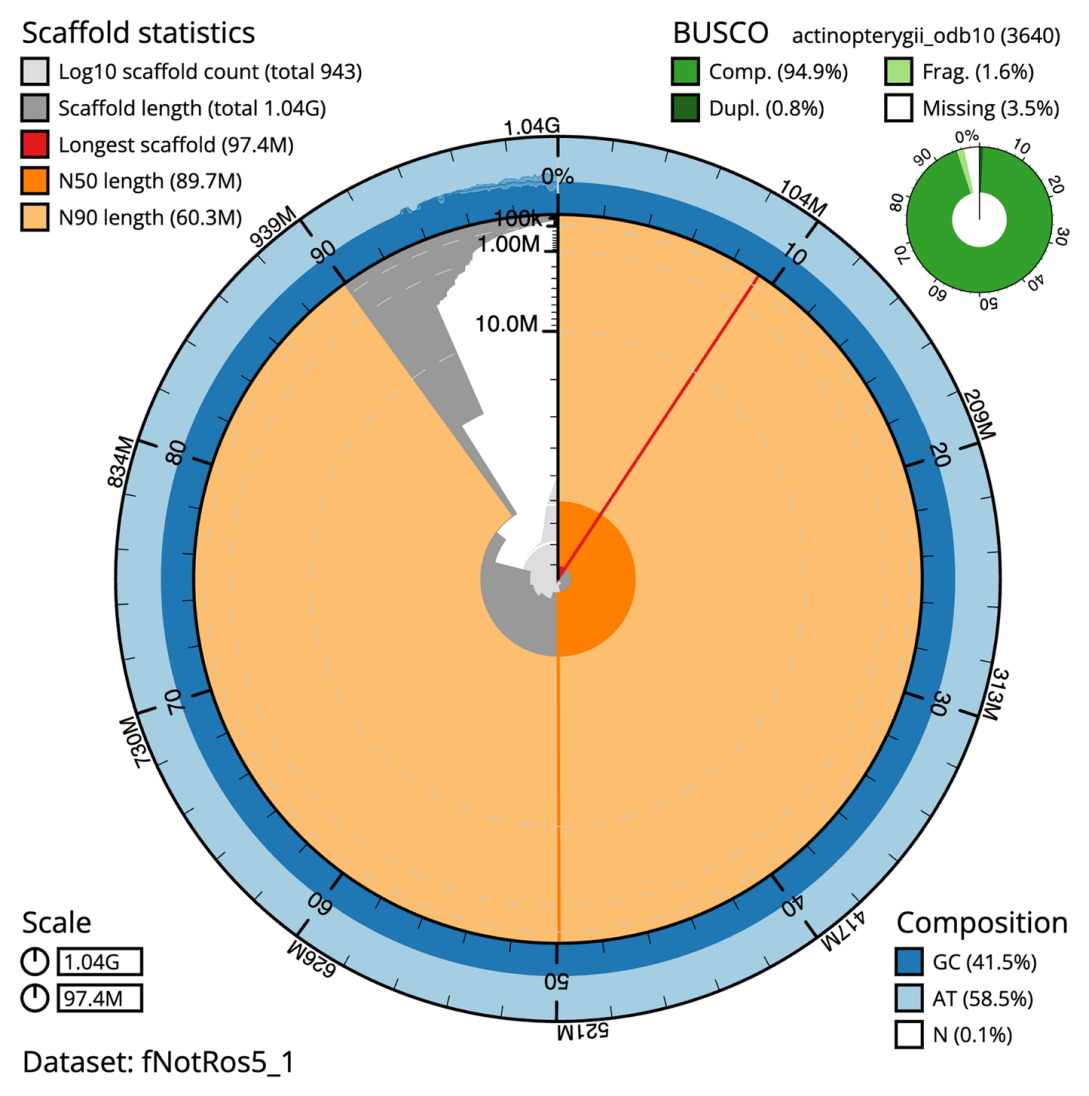
Genome assembly of *Notothenia rossii*, fNotRos5.1: metrics. The BlobToolKit snail plot shows N50 metrics and BUSCO gene completeness. The main plot is divided into 1,000 size-ordered bins around the circumference with each bin representing 0.1% of the 1,042,906,029 bp assembly. The distribution of scaffold lengths is shown in dark grey with the plot radius scaled to the longest scaffold present in the assembly (97,391,511 bp, shown in red). Orange and pale-orange arcs show the N50 and N90 scaffold lengths (89,684,120 and 60,281,083 bp), respectively. The pale grey spiral shows the cumulative scaffold count on a log scale with white scale lines showing successive orders of magnitude. The blue and pale-blue area around the outside of the plot shows the distribution of GC, AT and N percentages in the same bins as the inner plot. A summary of complete, fragmented, duplicated and missing BUSCO genes in the actinopterygii_odb10 set is shown in the top right. An interactive version of this figure is available at https://blobtoolkit.genomehubs.org/view/fNotRos5_1/dataset/fNotRos5_1/snail.

**Figure 3.**
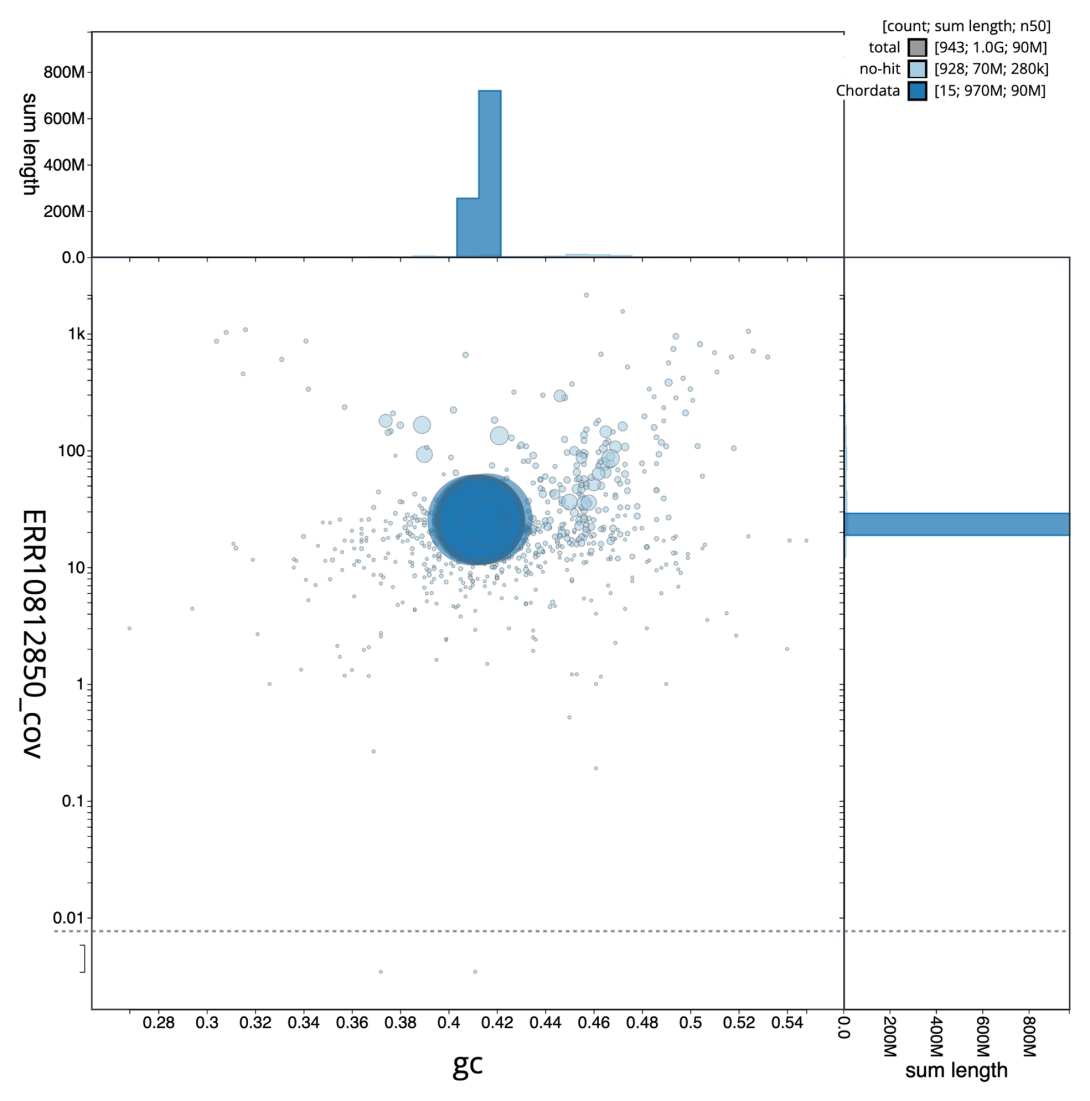
Genome assembly of *Notothenia rossii*, fNotRos5.1: BlobToolKit GC-coverage plot. Sequences are coloured by phylum. Circles are sized in proportion to sequence length. Histograms show the distribution of sequence length sum along each axis. An interactive version of this figure is available at https://blobtoolkit.genomehubs.org/view/fNotRos5_1/dataset/fNotRos5_1/blob.

**Figure 4.**
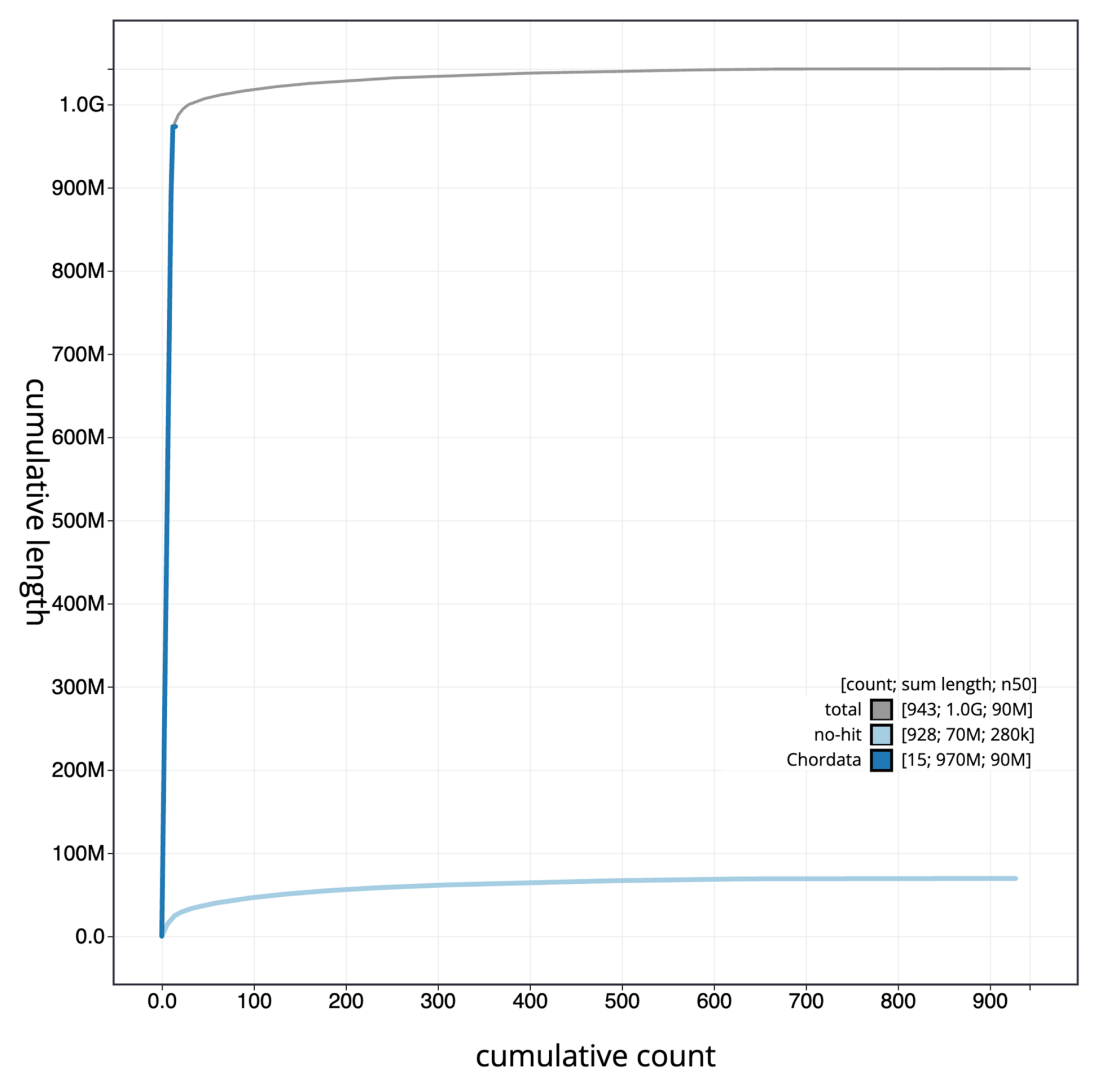
Genome assembly of *Notothenia rossii*, fNotRos5.1: BlobToolKit cumulative sequence plot. The grey line shows cumulative length for all sequences. Coloured lines show cumulative lengths of sequences assigned to each phylum using the buscogenes taxrule. An interactive version of this figure is available at https://blobtoolkit.genomehubs.org/view/fNotRos5_1/dataset/fNotRos5_1/cumulative.

**Figure 5.**
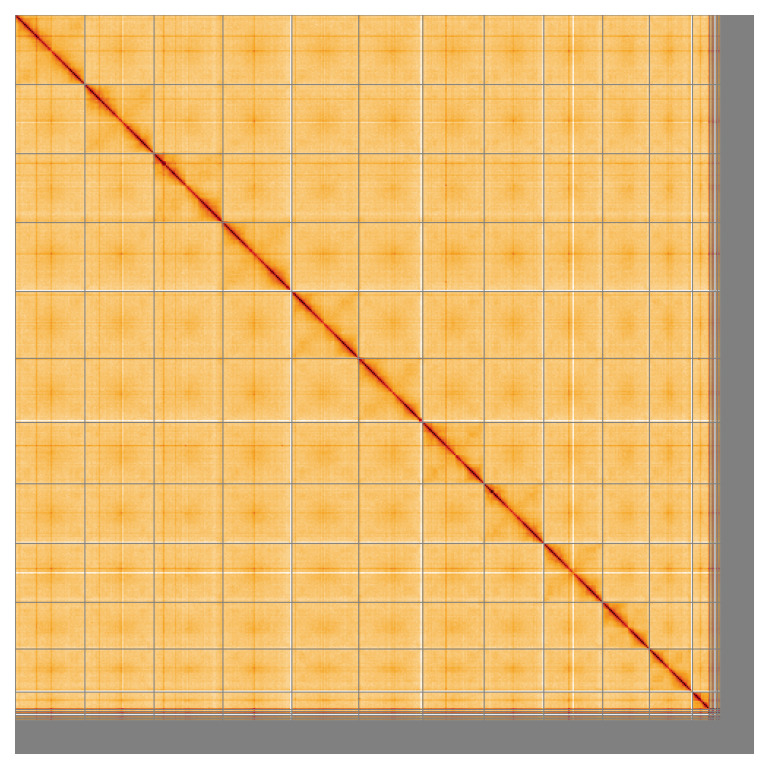
Genome assembly of *Notothenia rossii*, fNotRos5.1: Hi-C contact map of the fNotRos5.1 assembly, visualised using HiGlass. Chromosomes are shown in order of size from left to right and top to bottom. An interactive version of this figure may be viewed at https://genome-note-higlass.tol.sanger.ac.uk/l/?d=Fz2tF8wBS-qgF_W_5Ixaqw.

The final assembly has a total length of 1,042.9 Mb in 942 sequence scaffolds with a scaffold N50 of 89.7 Mb ( Table 1). The snail plot in Figure 2 provides a summary of the assembly statistics, while the distribution of assembly scaffolds on GC proportion and coverage is shown in Figure 3. The cumulative assembly plot in Figure 4 shows curves for subsets of scaffolds assigned to different phyla. Most (93.47%) of the assembly sequence was assigned to 12 chromosomal-level scaffolds. Chromosome-scale scaffolds confirmed by the Hi-C data are named in order of size ( Figure 5; Table 2). While not fully phased, the assembly deposited is of one haplotype. Contigs corresponding to the second haplotype have also been deposited. The mitochondrial genome was also assembled and can be found as a contig within the multifasta file of the genome submission.

**Table 1.** Genome data for *Notothenia rossii*, fNotRos5.1.

Project accession data		
Assembly identifier	fNotRos5.1	
Species	*Notothenia rossii*	
Specimen	fNotRos5	
NCBI taxonomy ID	101497	
BioProject	PRJEB59163	
BioSample ID	SAMEA12815441	
Isolate information	fNotRos5 (DNA, Hi-C and RNA sequencing)	
Assembly metrics *		*Benchmark*
Consensus quality (QV)	53.7	*≥ 50*
*k*-mer completeness	99.98%	*≥ 95%*
BUSCO **	C:94.9%[S:94.1%,D:0.8%], F:1.6%,M:3.5%,n:3,640	*C ≥ 95%*
Percentage of assembly mapped to chromosomes	93.47%	*≥ 95%*
Sex chromosomes	None	*localised homologous pairs*
Organelles	Mitochondrial genome: 21.68 kb	*complete single alleles*
Raw data accessions		
PacificBiosciences SEQUEL II	ERR10812850	
Hi-C Illumina	ERR10802490	
PolyA RNA-Seq Illumina	ERR11641121	
Genome assembly		
Assembly accession	GCA_949606895.1	
*Accession of alternate haplotype*	GCA_949606865.1	
Span (Mb)	1,042.9	
Number of contigs	5,100	
Contig N50 length (Mb)	0.4	
Number of scaffolds	942	
Scaffold N50 length (Mb)	89.7	
Longest scaffold (Mb)	97.77	
Genome annotation		
Number of protein-coding genes	24,432	
Number of non-coding genes	9,562	
Number of gene transcripts	68,226	

* Assembly metric benchmarks are adapted from column VGP-2020 of “Table 1: Proposed standards and metrics for defining genome assembly quality” from Rhie *et al.* (2021). ** BUSCO scores based on the actinopterygii_odb10 BUSCO set using version 5.3.2. C = complete [S = single copy, D = duplicated], F = fragmented, M = missing, n = number of orthologues in comparison. A full set of BUSCO scores is available at https://blobtoolkit.genomehubs.org/view/fNotRos5_1/dataset/fNotRos5_1/busco.

**Table 2.** Chromosomal pseudomolecules in the genome assembly of *Notothenia rossii*, fNotRos5.

INSDC accession	Chromosome	Length (Mb)	GC%
OX449111.1	1	97.39	41.0
OX449112.1	2	96.85	41.0
OX449113.1	3	96.53	41.5
OX449114.1	4	96.43	41.5
OX449115.1	5	93.75	41.5
OX449116.1	6	89.68	41.5
OX449117.1	7	85.89	41.5
OX449118.1	8	83.62	41.5
OX449119.1	9	82.49	41.5
OX449120.1	10	65.36	41.5
OX449121.1	11	60.28	41.0
OX449122.1	12	24.47	41.5
OX449123.1	MT	0.02	46.0

The estimated Quality Value (QV) of the final assembly is 53.7 with *k*-mer completeness of 99.98%, and the assembly has a BUSCO v5.3.2 completeness of 94.9% (single = 94.1%, duplicated = 0.8%), using the actinopterygii_odb10 reference set ( *n* = 3,640).

Metadata for specimens, barcode results, spectra estimates, sequencing runs, contaminants and pre-curation assembly statistics are given at https://links.tol.sanger.ac.uk/species/101497.

## Genome annotation report

The *Notothenia rossii* genome assembly (GCA_949606895.1) was annotated at the European Bioinformatics Institute (EBI) on Ensembl Rapid Release. The resulting annotation includes 66,226 transcribed mRNAs from 24,432 protein-coding and 9,562 non-coding genes ( Table 1; https://rapid.ensembl.org/Notothenia_rossii_GCA_949606895.1/Info/Index).

## Methods

### Sample acquisition and nucleic acid extraction

A juvenile specimen of *Notothenia rossii* was collected (BioSample: SAMEA12815441, fNotRos5) from the area of the Antarctic Polar Front near S. Georgia on 16 December 2019. The individual was captured using a Neuston net and preserved in a tube in the a –80°C freezer until processing. The specimen was collected by Iliana Bista and identified by Martin Collins.

The workflow for high molecular weight (HMW) DNA extraction at the Wellcome Sanger Institute (WSI) includes a sequence of core procedures: sample preparation; sample homogenisation, DNA extraction, fragmentation, and clean-up. In sample preparation, the fNotRos5 sample was weighed and dissected on dry ice ( Jay *et al.*, 2023).

For sample homogenisation, tissue was cryogenically disrupted using the Covaris cryoPREP ^®^ Automated Dry Pulverizer ( Narváez-Gómez *et al.*, 2023). HMW DNA was extracted using the Automated MagAttract v1 protocol ( Sheerin *et al.*, 2023). DNA was sheared into an average fragment size of 12–20 kb in a Megaruptor 3 system with speed setting 30 ( Todorovic *et al.*, 2023). Sheared DNA was purified by solid-phase reversible immobilisation ( Strickland *et al.*, 2023): in brief, the method employs a 1.8X ratio of AMPure PB beads to sample to eliminate shorter fragments and concentrate the DNA. The concentration of the sheared and purified DNA was assessed using a Nanodrop spectrophotometer and Qubit Fluorometer and Qubit dsDNA High Sensitivity Assay kit. Fragment size distribution was evaluated by running the sample on the FemtoPulse system.

RNA was extracted from tissue of fNotRos5 in the Tree of Life Laboratory at the WSI using the RNA Extraction: Automated MagMax™ *mir*Vana protocol ( do Amaral *et al.*, 2023). The RNA concentration was assessed using a Nanodrop spectrophotometer and a Qubit Fluorometer using the Qubit RNA Broad-Range Assay kit. Analysis of the integrity of the RNA was done using the Agilent RNA 6000 Pico Kit and Eukaryotic Total RNA assay.

Protocols developed by the WSI Tree of Life laboratory are publicly available on protocols.io ( Denton *et al.*, 2023).

### Sequencing

Pacific Biosciences HiFi circular consensus DNA sequencing libraries were constructed according to the manufacturers’ instructions. Poly(A) RNA-Seq libraries were constructed using the NEB Ultra II RNA Library Prep kit. DNA and RNA sequencing was performed by the Scientific Operations core at the WSI on Pacific Biosciences SEQUEL II (HiFi) and Illumina NovaSeq 6000 (RNA-Seq) instruments. Hi-C data were also generated from tissue of fNotRos5 using the Arima2 kit and sequenced on the Illumina NovaSeq 6000 instrument.

### Genome assembly, curation and evaluation

Assembly was carried out with Hifiasm ( Cheng *et al.*, 2021) and haplotypic duplication was identified and removed with purge_dups ( Guan *et al.*, 2020). The assembly was then scaffolded with Hi-C data ( Rao *et al.*, 2014) using YaHS ( Zhou *et al.*, 2023). The assembly was checked for contamination and corrected as described previously ( Howe *et al.*, 2021). Manual curation was performed using HiGlass ( Kerpedjiev *et al.*, 2018) and PretextView ( Harry, 2022). The mitochondrial genome was assembled using MitoHiFi ( Uliano-Silva *et al.*, 2023), which runs MitoFinder ( Allio *et al.*, 2020) or MITOS ( Bernt *et al.*, 2013) and uses these annotations to select the final mitochondrial contig and to ensure the general quality of the sequence.

A Hi-C map for the final assembly was produced using bwa-mem2 ( Vasimuddin *et al.*, 2019) in the Cooler file format ( Abdennur & Mirny, 2020). To assess the assembly metrics, the *k*-mer completeness and QV consensus quality values were calculated in Merqury ( Rhie *et al.*, 2020). This work was done using Nextflow ( Di Tommaso *et al.*, 2017) DSL2 pipelines “sanger-tol/readmapping” ( Surana *et al.*, 2023a) and “sanger-tol/genomenote” ( Surana *et al.*, 2023b). The genome was analysed within the BlobToolKit environment ( Challis *et al.*, 2020) and BUSCO scores ( Manni *et al.*, 2021; Simão *et al.*, 2015) were calculated.

Table 3 contains a list of relevant software tool versions and sources.

**Table 3.** Software tools: versions and sources.

Software tool	Version	Source
BlobToolKit	4.1.7	https://github.com/blobtoolkit/blobtoolkit
BUSCO	5.3.2	https://gitlab.com/ezlab/busco
Hifiasm	0.16.1-r375	https://github.com/chhylp123/hifiasm
HiGlass	1.11.6	https://github.com/higlass/higlass
Merqury	MerquryFK	https://github.com/thegenemyers/MERQURY.FK
MitoHiFi	2	https://github.com/marcelauliano/MitoHiFi
PretextView	0.2	https://github.com/wtsi-hpag/PretextView
purge_dups	1.2.3	https://github.com/dfguan/purge_dups
sanger-tol/ genomenote	v1.0	https://github.com/sanger-tol/genomenote
sanger-tol/ readmapping	1.1.0	https://github.com/sanger-tol/readmapping/tree/1.1.0
YaHS	1.2a	https://github.com/c-zhou/yahs

### Genome annotation

The Ensembl Genebuild annotation system ( Aken *et al.*, 2016) was used to generate annotation for the *Notothenia rossii* assembly (GCA_949606895.1) in Ensembl Rapid Release at the EBI. Annotation was created primarily through alignment of transcriptomic data to the genome, with gap filling via protein-to-genome alignments of a select set of proteins from UniProt ( UniProt Consortium, 2019).

### Wellcome Sanger Institute – Legal and Governance

The materials that have contributed to this genome note have been supplied by a Tree of Life collaborator. The Wellcome Sanger Institute employs a process whereby due diligence is carried out proportionate to the nature of the materials themselves, and the circumstances under which they have been/are to be collected and provided for use. The purpose of this is to address and mitigate any potential legal and/or ethical implications of receipt and use of the materials as part of the research project, and to ensure that in doing so we align with best practice wherever possible.

The overarching areas of consideration are:

• Ethical review of provenance and sourcing of the material

• Legality of collection, transfer and use (national and international)

Each transfer of samples is undertaken according to a Research Collaboration Agreement or Material Transfer Agreement entered into by the Tree of Life collaborator, Genome Research Limited (operating as the Wellcome Sanger Institute) and in some circumstances other Tree of Life collaborators.

## Data Availability

European Nucleotide Archive: *Notothenia rossii* (marbled rockcod). Accession number PRJEB59163; https://identifiers.org/ena.embl/PRJEB59163 ( Wellcome Sanger Institute, 2023). The genome sequence is released openly for reuse. The *Notothenia rossii* genome sequencing initiative is part of the Vertebrate Genomes Project. All raw sequence data and the assembly have been deposited in INSDC databases. Raw data and assembly accession identifiers are reported in Table 1.

## Author information

Members of the Wellcome Sanger Institute Tree of Life Management, Samples and Laboratory team are listed here: https://doi.org/10.5281/zenodo.10066175.

Members of Wellcome Sanger Institute Scientific Operations: Sequencing Operations are listed here: https://doi.org/10.5281/zenodo.10043364.

Members of the Wellcome Sanger Institute Tree of Life Core Informatics team are listed here: https://doi.org/10.5281/zenodo.10066637.

Members of the Tree of Life Core Informatics collective are listed here: https://doi.org/10.5281/zenodo.5013541.

## References

[ref-1] Abdennur N Mirny LA : Cooler: scalable storage for Hi-C data and other genomically labeled arrays. *Bioinformatics.* 2020;36(1):311–316. 10.1093/bioinformatics/btz540 31290943 PMC8205516

[ref-2] Aken BL Ayling S Barrell D : The Ensembl gene annotation system. *Database (Oxford).* 2016;2016: baw093. 10.1093/database/baw093 27337980 PMC4919035

[ref-3] Allio R Schomaker-Bastos A Romiguier J : MitoFinder: efficient automated large-scale extraction of mitogenomic data in target enrichment phylogenomics. *Mol Ecol Resour.* 2020;20(4):892–905. 10.1111/1755-0998.13160 32243090 PMC7497042

[ref-4] Bernt M Donath A Jühling F : MITOS: improved *de novo* metazoan mitochondrial genome annotation. *Mol Phylogenet Evol.* 2013;69(2):313–319. 10.1016/j.ympev.2012.08.023 22982435

[ref-5] Bista I Wood JMD Desvignes T : Genomics of cold adaptations in the Antarctic notothenioid fish radiation. *Nat Commun.* 2023;14(1): 3412. 10.1038/s41467-023-38567-6 37296119 PMC10256766

[ref-6] Burchet M : The life cycle of *Notothenia rossii* from South Georgia. *Br Antarct Surv Bull.* 1983;61:71–73. Reference Source

[ref-50] Calì F Riginella E La Mesa M : Life history traits of *Notothenia rossii* and *N. coriiceps* along the southern Scotia Arc. *Polar Biol.* 2017;40:1409–1423. 10.1007/s00300-016-2066-z

[ref-7] Challis R Richards E Rajan J : BlobToolKit - interactive quality assessment of genome assemblies. *G3 (Bethesda).* 2020;10(4):1361–1374. 10.1534/g3.119.400908 32071071 PMC7144090

[ref-8] Chen L DeVries AL Cheng CH : Evolution of antifreeze glycoprotein gene from a trypsinogen gene in Antarctic notothenioid fish. *Proc Natl Acad Sci U S A.* 1997;94(8):3811–3816. 10.1073/pnas.94.8.3811 9108060 PMC20523

[ref-9] Cheng H Concepcion GT Feng X : Haplotype-resolved *de novo* assembly using phased assembly graphs with hifiasm. *Nat Methods.* 2021;18(2):170–175. 10.1038/s41592-020-01056-5 33526886 PMC7961889

[ref-10] Denton A Yatsenko H Jay J : Sanger Tree of Life wet laboratory protocol collection V.1. *protocols.io.* 2023. 10.17504/protocols.io.8epv5xxy6g1b/v1

[ref-11] DeWitt HH Heemstra PC Gon O : Nototheniidae.In: Gon, O. and Heemstra, P. C. (eds.) *Fishes of the Southern Ocean.* 1990;279–331.

[ref-12] Di Tommaso P Chatzou M Floden EW : Nextflow enables reproducible computational workflows. *Nat Biotechnol.* 2017;35(4):316–319. 10.1038/nbt.3820 28398311

[ref-13] do Amaral RJV Bates A Denton A : Sanger Tree of Life RNA extraction: automated MagMax ^TM^ mirVana. *protocols.io.* 2023. 10.17504/protocols.io.6qpvr36n3vmk/v1

[ref-14] Eastman JT : The nature of the diversity of Antarctic fishes. *Polar Biol.* 2005;28(2):93–107. 10.1007/s00300-004-0667-4

[ref-15] Eastman JT Eakin RR : Checklist of the species of notothenioid fishes. *Antarct Sci.* 2021;33(3):273–280. 10.1017/S0954102020000632

[ref-16] Guan D McCarthy SA Wood J : Identifying and removing haplotypic duplication in primary genome assemblies. *Bioinformatics.* 2020;36(9):2896–2898. 10.1093/bioinformatics/btaa025 31971576 PMC7203741

[ref-17] Harry E : PretextView (Paired REad TEXTure Viewer): a desktop application for viewing pretext contact maps. 2022; [Accessed 19 October 2022]. Reference Source

[ref-18] Hollyman PR Hill SL Laptikhovsky VV : A long road to recovery: dynamics and ecology of the marbled rockcod ( *Notothenia rossii*, family: Nototheniidae) at South Georgia, 50 years after overexploitation. *ICES J Mar Sci.* 2021;78(8):2745–2756. 10.1093/icesjms/fsab150

[ref-19] Howe K Chow W Collins J : Significantly improving the quality of genome assemblies through curation. *GigaScience.* 2021;10(1): giaa153. 10.1093/gigascience/giaa153 33420778 PMC7794651

[ref-20] Jay J Yatsenko H Narváez-Gómez JP : Sanger Tree of Life Sample preparation: triage and dissection. *protocols.io.* 2023. 10.17504/protocols.io.x54v9prmqg3e/v1

[ref-21] Kerpedjiev P Abdennur N Lekschas F : HiGlass: web-based visual exploration and analysis of genome interaction maps. *Genome Biol.* 2018;19(1): 125. 10.1186/s13059-018-1486-1 30143029 PMC6109259

[ref-22] Kock KH Belchier M Jones CD : Is the attempt to estimate the biomass of Antarctic fish from a multi-species survey appropriate for all targeted species? *Notothenia rossii* in the Atlantic Ocean sector – revisited. *CCAMLR Science.* 2004;11:141–153. Reference Source

[ref-23] Manni M Berkeley MR Seppey M : BUSCO update: novel and streamlined workflows along with broader and deeper phylogenetic coverage for scoring of eukaryotic, prokaryotic, and viral genomes. *Mol Biol Evol.* 2021;38(10):4647–4654. 10.1093/molbev/msab199 34320186 PMC8476166

[ref-24] Matschiner M Hanel R Salzburger W : On the origin and trigger of the notothenioid adaptive radiation. *PLoS One.* 2011;6(4): e18911. 10.1371/journal.pone.0018911 21533117 PMC3078932

[ref-25] Narváez-Gómez JP Mbye H Oatley G : Sanger Tree of Life Sample homogenisation: covaris cryoPREP ^®^ automated dry pulverizer V.1. *protocols.io.* 2023. 10.17504/protocols.io.eq2lyjp5qlx9/v1

[ref-26] Rao SSP Huntley MH Durand NC : A 3D map of the human genome at kilobase resolution reveals principles of chromatin looping. *Cell.* 2014;159(7):1665–1680. 10.1016/j.cell.2014.11.021 25497547 PMC5635824

[ref-27] Rhie A McCarthy SA Fedrigo O : Towards complete and error-free genome assemblies of all vertebrate species. *Nature.* 2021;592(7856):737–746. 10.1038/s41586-021-03451-0 33911273 PMC8081667

[ref-28] Rhie A Walenz BP Koren S : Merqury: reference-free quality, completeness, and phasing assessment for genome assemblies. *Genome Biol.* 2020;21(1): 245. 10.1186/s13059-020-02134-9 32928274 PMC7488777

[ref-29] Sheerin E Sampaio F Oatley G : Sanger Tree of Life HMW DNA extraction: automated MagAttract v.1. *protocols.io.* 2023. 10.17504/protocols.io.x54v9p2z1g3e/v1

[ref-30] Simão FA Waterhouse RM Ioannidis P : BUSCO: assessing genome assembly and annotation completeness with single-copy orthologs. *Bioinformatics.* 2015;31(19):3210–3212. 10.1093/bioinformatics/btv351 26059717

[ref-31] Strickland M Cornwell C Howard C : Sanger Tree of Life fragmented DNA clean up: manual SPRI. *protocols.io.* 2023. 10.17504/protocols.io.kxygx3y1dg8j/v1

[ref-32] Surana P Muffato M Qi G : sanger-tol/readmapping: sanger-tol/readmapping v1.1.0 - Hebridean Black (1.1.0). *Zenodo.* 2023a. 10.5281/zenodo.7755669

[ref-33] Surana P Muffato M Sadasivan Baby C : sanger-tol/genomenote (v1.0.dev). *Zenodo.* 2023b. 10.5281/zenodo.6785935

[ref-34] Todorovic M Sampaio F Howard C : Sanger Tree of Life HMW DNA fragmentation: Diagenode Megaruptor® 3 for PacBio HiFi. *protocols.io.* 2023. 10.17504/protocols.io.8epv5x2zjg1b/v1

[ref-35] Uliano-Silva M Ferreira JGRN Krasheninnikova K : MitoHiFi: a python pipeline for mitochondrial genome assembly from PacBio high fidelity reads. *BMC Bioinformatics.* 2023;24(1): 288. 10.1186/s12859-023-05385-y 37464285 PMC10354987

[ref-36] UniProt Consortium: UniProt: a worldwide hub of protein knowledge. *Nucleic Acids Res.* 2019;47(D1):D506–D515. 10.1093/nar/gky1049 30395287 PMC6323992

[ref-37] Vasimuddin M Misra S Li H : Efficient architecture-aware acceleration of BWA-MEM for multicore systems. In: *2019 IEEE International Parallel and Distributed Processing Symposium (IPDPS)*. IEEE,2019;314–324. 10.1109/IPDPS.2019.00041

[ref-38] Wellcome Sanger Institute: The genome sequence of the marbled rockcod, *Notothenia rossii* Richardson, 1844. European Nucleotide Archive, [dataset], accession number PRJEB59163,2023.

[ref-39] Young E Rock J Meredith M : Physical and behavioural influences on larval fish retention: contrasting patterns in two Antarctic fishes. *Mar Ecol Prog Ser.* 2012;465:201–215. 10.3354/meps09908

[ref-40] Zhou C McCarthy SA Durbin R : YaHS: yet another Hi-C scaffolding tool. *Bioinformatics.* 2023;39(1): btac808. 10.1093/bioinformatics/btac808 36525368 PMC9848053

