# Supplementary material for: The genome sequence of the marbled rockcod, Notothenia rossii Richardson, 1844
Source: Wellcome Open Res. 2024 Apr 24;9:227. [Version 1] doi: 10.12688/wellcomeopenres.21270.1 (PMC11362733; doi:10.12688/wellcomeopenres.21270.1)
Supplement: Peer review history

# Reviewer report: Reviewer response for version 1

**DOI:** 10.21956/wellcomeopenres.23525.r87162

**Published:** 2024-08-29

**Authors:** Hyun Park

**Affiliations:** 1 Korea University, Seoul, South Korea

This work intended to provide high quality Antarctic marbled rockcod through incorporation of Pacbio Hifi and Hi-C sequencing data. This paper is interesting and establish meaningful groundwork for advancing Antarctic fish studies.

 What is the sex of the sample used, male or female?

 Which organ did you use to extract DNA and RNA?

 It would be better to add which species of protein sequence was used for annotation.

Are sufficient details of methods and materials provided to allow replication by others?

Yes

Is the rationale for creating the dataset(s) clearly described?

Yes

Are the datasets clearly presented in a useable and accessible format?

Yes

Are the protocols appropriate and is the work technically sound?

Yes

Reviewer Expertise:

Genomics

I confirm that I have read this submission and believe that I have an appropriate level of expertise to confirm that it is of an acceptable scientific standard.

# Reviewer report: Reviewer response for version 1

**DOI:** 10.21956/wellcomeopenres.23525.r86179

**Published:** 2024-07-23

**Authors:** Chenhong Li

**Affiliations:** 1 Shanghai Universities Key Laboratory of Marine Animal Taxonomy and Evolution, Shanghai Ocean University, Shanghai, China

The work is well done. Data collected and assembly and annotation methods all followed lasted high standard. Introduction is well writen. I only have two suggestions: add a phylogenetic tree showing the relationship of the group and relatives and note which ones have genomes available. This may suggests what need to be done to fill the gaps; comparison the statistics between the current genome assembly and the previous one.

Are sufficient details of methods and materials provided to allow replication by others?

Yes

Is the rationale for creating the dataset(s) clearly described?

Yes

Are the datasets clearly presented in a useable and accessible format?

Yes

Are the protocols appropriate and is the work technically sound?

Yes

Reviewer Expertise:

Phylogenetics, comparative genetics

I confirm that I have read this submission and believe that I have an appropriate level of expertise to confirm that it is of an acceptable scientific standard.

# Reviewer report: Reviewer response for version 1

**DOI:** 10.21956/wellcomeopenres.23525.r86185

**Published:** 2024-07-12

**Authors:** Alejandro S. Mechaly

**Affiliations:** 1 Instituto de Investigaciones en Biodiversidad y Biotecnología (INBIOTEC-CONICET), Mar del Plata, Argentina

The manuscript titled "The genome sequence of the marbled rockcod, *Notothenia rossii* Richardson, 1844" presents a comprehensive genome assembly and annotation of the marbled rockcod, *Notothenia rossii* . The assembly spans 1,042.9 megabases and includes 12 chromosomal pseudomolecules, along with the mitochondrial genome. Gene annotation on Ensembl identified a total of 24,432 protein-coding genes.

 The manuscript presents a significant advancement in the genomics data of *Notothenia rossii* , offering a comprehensive genome assembly and annotation. It serves as a critical resource for advancing research in comparative genomics and evolutionary biology within Perciformes. To enhance its scientific impact, I recommend the authors consider incorporating additional comparative analyses.

**Comments:**

**INTRODUCTION**

 The introduction lacks coherence and flow. It transitions abruptly from general fish biology to mentioning antifreeze genes ( *afgp* ) without clear context. Subsequently, it briefly touches on the fishery importance and overexploitation issues, but fails to articulate the specific goals of obtaining the genome. What studies are envisioned with an available genome? Why sequence a higher-quality genome than previously achieved? What new insights can be gleaned at the chromosomal level that were not possible before? Additionally, the contribution of the mitochondrial genome is not clearly articulated.

 The manuscript would benefit significantly from a more structured development and utilization of the sequenced genome. If the aim is to study the antifreeze genes ( *afgp* ), comparative bioinformatics work with similar genes from other species should be included. For conservation studies, numerous comparative analyses could be conducted, which are not explored in the current manuscript. There is a clear need to outline the rationale behind obtaining a high-quality genome for this species.

**METHODOLOGY**

- The manuscript lacks crucial details regarding the methods for sampling and preserving fish specimens after sacrifice. Specifically, there is no information provided on how samples were taken and preserved for subsequent analyses. Furthermore, it is unclear whether the study received approval from an ethics committee. If an ethics committee approval was obtained, details regarding which committee granted approval (e.g., Institutional Animal Care and Use Committee, IACUC) should be included for transparency and compliance with ethical standards.
- The manuscript mentions a previous sequencing effort in the species but fails to discuss the sequencing methodologies employed or highlight any advancements or differences in sequencing technologies used in the current study.
- It is crucial to address why individuals of both sexes have not been sequenced. This omission is particularly significant because sequencing both sexes could reveal sex chromosomes, sex-determining genes, or allow for a more comprehensive integration of genomic data.
- Understanding the genomic basis of sex determination and potential sex-specific differences is essential for a thorough understanding of the species. Integrating data from both male and female individuals could provide insights into sex-linked genomic regions and contribute to broader evolutionary and biological studies. Therefore, I recommend the authors address these points to strengthen the manuscript's comprehensiveness and relevance to the field of genomics and evolutionary biology.
- The manuscript lacks essential information regarding the RNA extraction process, specifically which tissue(s) were utilized. It is crucial to clarify why these particular tissues were selected for RNA extraction.
- To enhance the manuscript's clarity and methodological transparency, it is recommended that the authors provide a detailed explanation of the tissues chosen for RNA extraction, including any biological or experimental justifications for their selection. This addition will strengthen the scientific rigor of the study and improve its reproducibility by facilitating a better understanding of the experimental design.

**RESULTS**

- Figures 3, 4, and 5 in the manuscript depict sequence quality and assembly metrics; however, there is limited subsequent analysis or discussion regarding the insights gained from this information. These figures are critical for evaluating the reliability and completeness of the genome assembly, but their potential to inform subsequent results or biological implications remains underexplored.
- To strengthen the manuscript, it is advisable to leverage the data presented in these figures to provide deeper insights into the genome's characteristics. For instance, discussions could include how assembly quality influences the identification of specific genomic features, comparative genomic analyses, or evolutionary implications within the species or related taxa. Additionally, linking these metrics with functional genomic data or highlighting specific genomic regions of interest would enhance the manuscript's scientific impact and relevance.

Addressing these points will ensure that the figures not only serve as quality assessments but also contribute substantively to the overall findings and interpretations presented in the manuscript.

- The study presents a genome assembly of the marbled rockcod, Notothenia rossii, spanning 1,042.9 megabases with scaffolded chromosomal pseudomolecules and a fully assembled mitochondrial genome of 21.68 kilobases. Gene annotation identified 24,432 protein-coding genes on Ensembl. While this assembly represents a comprehensive genomic dataset for Notothenia rossii, the manuscript falls short in leveraging this resource for broader comparative biological insights.
- To strengthen the manuscript, the authors should consider discussing potential applications of the genome assembly beyond basic gene annotation. Highlighting specific research questions that can be addressed with this dataset, such as adaptations to cold environments or evolutionary trajectories within Antarctic fish, would broaden its significance and appeal to a wider audience in the scientific community.

Are sufficient details of methods and materials provided to allow replication by others?

Partly

Is the rationale for creating the dataset(s) clearly described?

Partly

Are the datasets clearly presented in a useable and accessible format?

Partly

Are the protocols appropriate and is the work technically sound?

Partly

Reviewer Expertise:

Molecular Biology. Fish reproduction and Endocrinology. Genomics. Computational Biology

I confirm that I have read this submission and believe that I have an appropriate level of expertise to confirm that it is of an acceptable scientific standard, however I have significant reservations, as outlined above.
